# A dataset of study program availability in German higher education between 1971 and 1996

**DOI:** 10.1038/s41597-025-06052-y

**Published:** 2025-10-08

**Authors:** Boris Thome, Friederike Hertweck, Serife Yasar, Lukas Jonas, Stefan Conrad

**Affiliations:** 1 Heinrich-Heine University Düsseldorf, Chair of Databases and Information Systems, Düsseldorf, D-40225 Germany; 2 RWI – Leibniz Institute for Economic Research, Department for Labor Markets, Education and Population, Essen, D-45128 Germany; 3 Ruhr University Bochum, Chair of Empirical Economics, Bochum, D-44801 Germany

**Keywords:** Education, Sociology

## Abstract

Educational systems are dynamic. They shape human capital, technological and societal progress, and also economic growth. Higher education, in particular, fosters innovation, with varying fields of study contributing differently to this process. Yet, despite its importance, no dataset has previously documented the evolution of academic fields across higher education institutions in a specific country. Addressing this gap, we present the RWI-UNI-SUBJECTS^1^ dataset, the first extensive collection of study opportunities across German higher education institutions between 1971 and 1996. The dataset originates from annual study guides by the German Federal Employment Agency for high school students. To extract the data, a custom-developed computer vision algorithm was used. We further enriched the dataset with administrative codes for fields, institutions, and districts, enabling seamless integration with additional datasets, such as social security data, official student statistics, or the National Educational Panel Study (NEPS). Covering a total of 105,307 study programs between 1971 and 1996, RWI-UNI-SUBJECTS^1^ offers a valuable foundation for interdisciplinary research on education, innovation, and economic development.

## Background & Summary

Despite the central role of higher education in shaping research as well as societal and economic development, systematic data on the historical evolution of study programs across institutions and regions has remained remarkably scarce. This lack of data has constrained empirical research on the dynamics of educational supply and its broader implications for innovation, regional development, and labor markets. This gap is particularly striking given the significance of the period from the 1970s onwards that marked a phase of major expansion in higher education across much of the Western world. In Germany, as elsewhere, the 1970s and 1980s saw the foundation of numerous new institutions, the emergence of novel academic fields such as computer science, and a sharp rise in student enrollment driven by the baby boomer generation^2,3^.

RWI-UNI-SUBJECTS^1^ fills this substantial gap by delivering novel data on the evolution of higher education in general and specific study programs in particular. It covers the universe of all 105,307 different study programs at universities and universities of applied sciences in Germany between 1971 and 1996. The dataset is extracted from the official study guides entitled “Study and Career Choice”. These books were annually published by the German Federal Employment Agency to inform high school students about *all* post-secondary educational opportunities available in Germany^4–29^. Notably, while education in Germany is predominantly decentralized with each federal state overseeing its educational framework, the creation of the study guides was coordinated by a single central authority: the Commission for Educational Planning and Research Promotion (BLK). This institutional arrangement ensured the uniformity and comparability of the information across all states and facilitated the development of a standardized resource that encompassed all institutions, study programs, and practical aspects on student housing and financial aid. The centralization of this effort by the BLK is particularly noteworthy, as it allowed for the systematic compilation and continuous updating of educational data on a national scale. It furthermore allowed for comparability over time, with full coverage extending from Western Germany prior to 1990 and later encompassing the reunified Germany.

To the best of our knowledge, only two datasets comparable to RWI-UNI-SUBJECTS exist. The first is the College Scorecard dataset from the US^30^, which provides comprehensive institution-level data from 1996 to 2023 and subject-level data from 2014 to 2020. It includes information on enrollment, financial aid, costs, debt, repayment, and post-graduation earnings. Additionally, it offers crosswalk files linking colleges’ identification codes (OPEID) with identifiers from the Integrated Postsecondary Education Data System (IPEDS UnitID). The second dataset is the Catalogue of First and Second Cycle Degree Programmes from the University of Bologna^31^, which contains detailed program data for the University of Bologna by academic year from 2004/2005 to 2024/2025. Neither dataset fully covers or substitutes for the RWI-UNI-SUBJECTS dataset, as both provide information on only a subsample of institutions, cover different periods, and do not address the years of higher education expansion at the end of the last century.

An essential component of the guides was a set of tables that indicated which institutions offered specific study programs. These tables, which serve as the main component of our dataset, were extracted using a custom-developed computer vision algorithm^32^, allowing for automated and precise data extraction. To enhance the dataset’s utility, we enriched it with administrative codes for institutions, fields, and districts. This enables seamless linkage with other administrative and survey data to facilitate research across a wide range of disciplines. The resulting dataset can be easily connected to social security data^33^, official higher education statistics^34^, or panel data such as the National Educational Panel Study^35^, the German Socio-Economic Panel^36^ or the DZHW Graduate Panel^37^ via these identifiers for institutions, study programs, and districts.

**Fig. 1:**
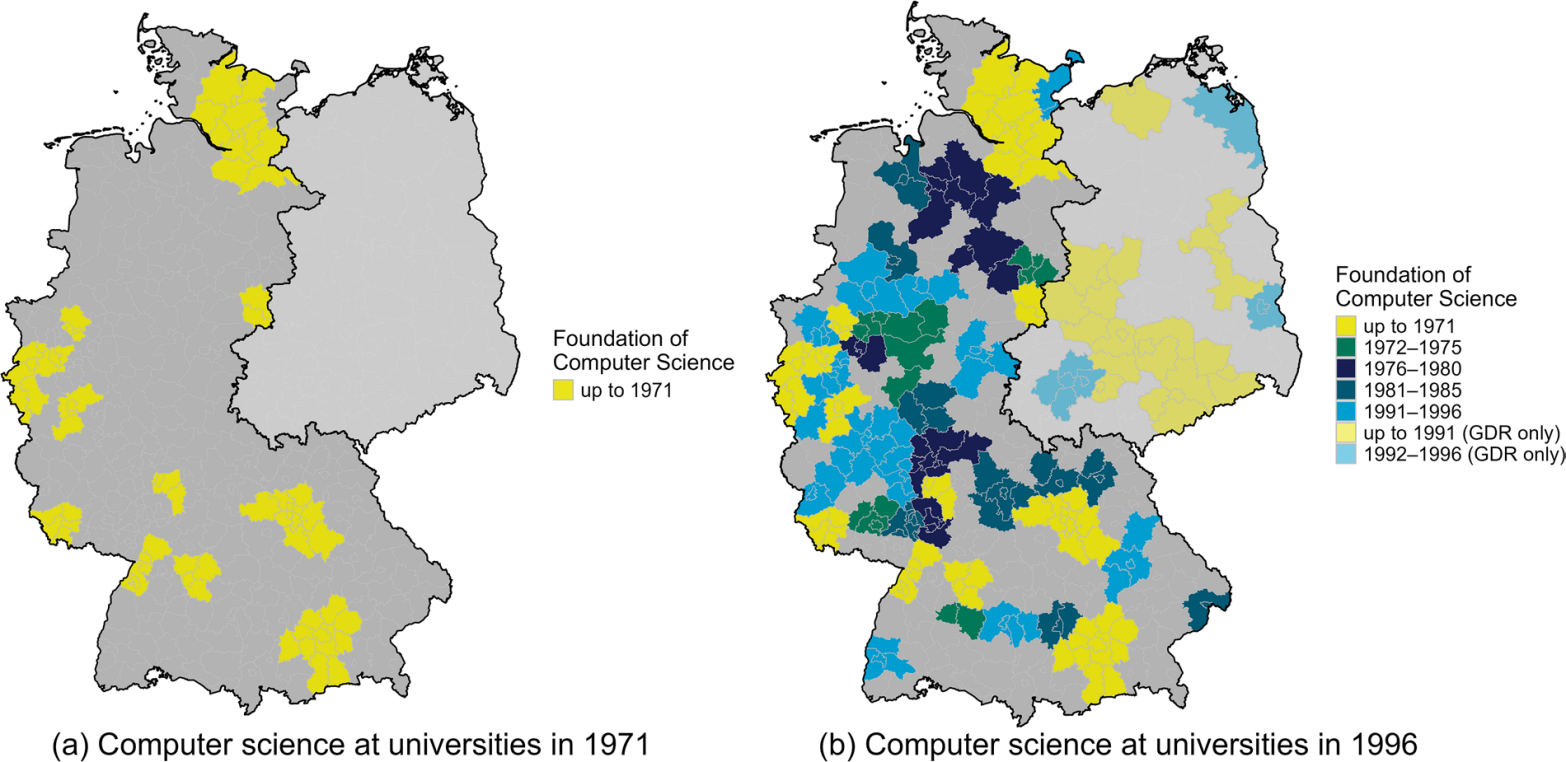
Expansion of computer science at universities across commuting zones. Note: Panel a and b provide an illustration of the evolution of the field computer science at universities across commuting zones over time. Panel a provides the availability of computer science at universities in 1971. Panel b illustrates the evolution of computer science at universities until 1996. Both panels are based on the RWI-UNI-SUBJECTS^1^ dataset linked to information on commuting zones^38^. Until 1990, the former German Democratic Republic (GDR) was not part of Germany, which is indicated by the light gray shades in both panels. Data about institutions in the former GDR is only available for the time after the German reunification.

Figure 1 illustrates the research potential of the dataset by tracing the spatial and temporal expansion of the field of computer science at universities across German commuting zones. For this purpose, the dataset was linked to official commuting zone classifications via the municipality codes^38^. Panel a shows that in 1971, the field was offered in only a few commuting zones across Germany. By 1996, computer science had expanded substantially (Panel b) with growth in these programs occurring mostly from the 1980s onwards. It becomes clear that some parts of Germany, such as the entire northwestern region, only gained access to computer science programs from the 1980s onwards. Several areas, however, still lacked such opportunities in 1996, as indicated by the gray-shaded zones in Fig. 1. This example highlights the dataset’s value for geographically and temporally disaggregated analyses of regional disparities and the diffusion of academic fields within the higher education system.

**Fig. 2:**
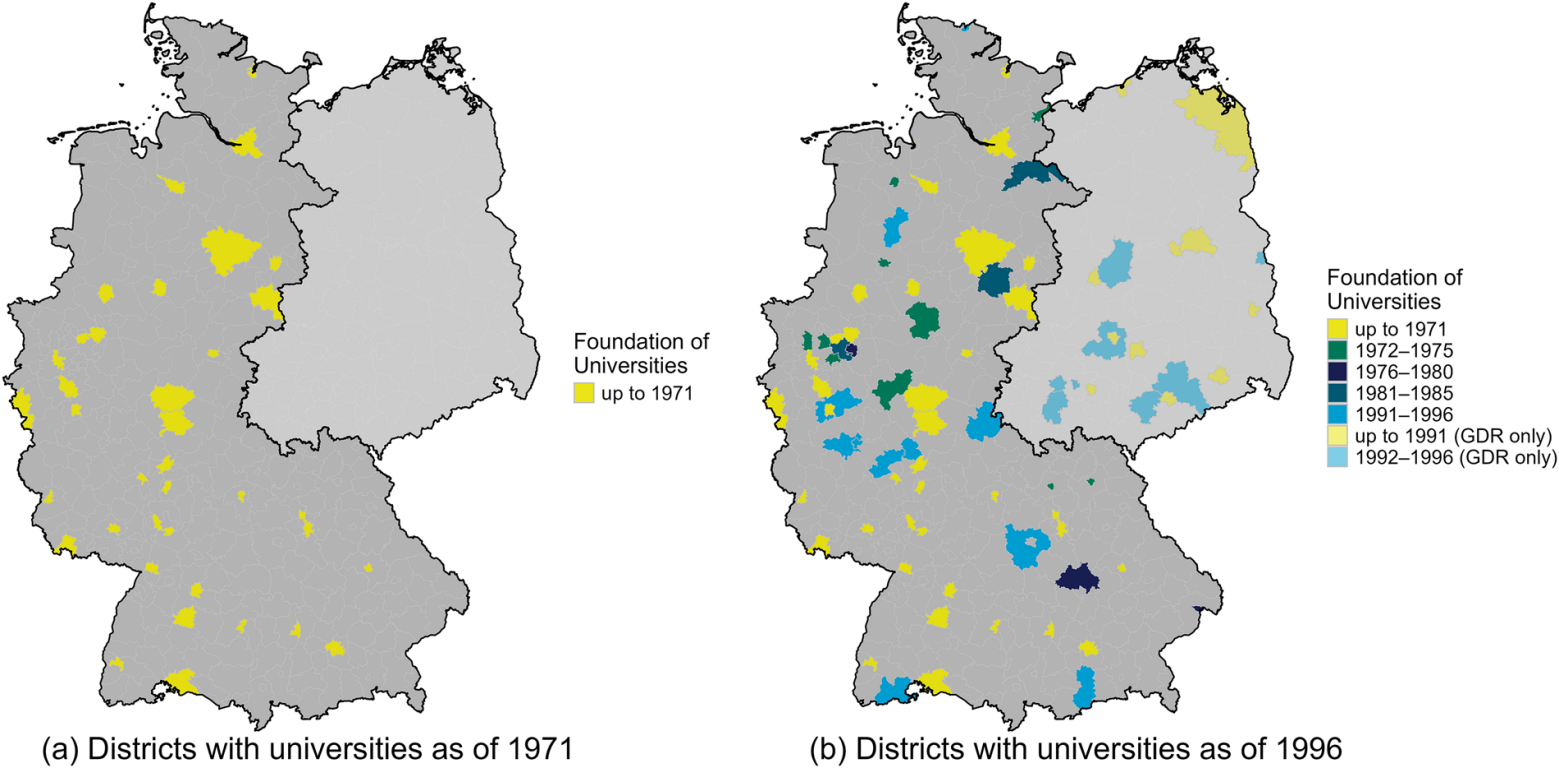
Expansion of universities across districts. Note: Until 1990, the former German Democratic Republic (GDR) was not part of Germany, which is indicated by the light gray shades in both panels. Panel a provides the availability of universities across districts in 1971. Data about institutions in the former GDR is only available for the time after the German reunification. Panel b illustrates the evolution of universities in Germany until 1996.

Similarly, Fig. 2 shows the district-level distribution of universities in general. The changes from Panel a to Panel b highlight two major expansion phases: the first wave in the 1970s, reflecting efforts to broaden access to higher education, and the second expansion in the early 1990s. Figure 2 illustrates how the dataset supports analyses of institutional change and regional access to higher education. It has already been used to study the impact of new university foundations on local educational choices^39^.

The dataset also enables the analysis of program structures and allows for the distinction between different types of study programs. The variable study_type captures the structural and administrative categorization of study programs within the German higher education system during the observation period. It distinguishes between full study programs *(in German: Vollstudium)*, minor subjects, specialization tracks, and advanced studies, as well as their corresponding admission types (e.g., admission-restricted) and recommended or required starting terms. The differentiation reflects program accessibility – such as whether programs are open to new students, require specific entry terms, or are planned to be discontinued.

Overall, this dataset offers a unique foundation for investigating the long-term dynamics in higher education, characterized by broad coverage and standardized classifications. Despite ending in 1996, the dataset is a valuable resource for understanding how institutional expansion, program diversification, and reforms shaped modern education systems. Its historical scope enables scholars to investigate how higher education responded to changing societal and labor market demands, economic developments, and the emergence of the information age in a globalized economy. The dataset’s standardized format further supports comparative research across time and regions and can reveal links between fields of study, regional development, industrial specialization, and workforce skills. Although more recent data is neither as easily accessible nor standardized, this dataset still offers critical insights into the lasting influence of past educational, regional, and industrial policies and their ongoing impact on current challenges in higher education and society. It has already been utilized in a study examining the educational expansion in Germany^39^.

## Methods

**Fig. 3:**
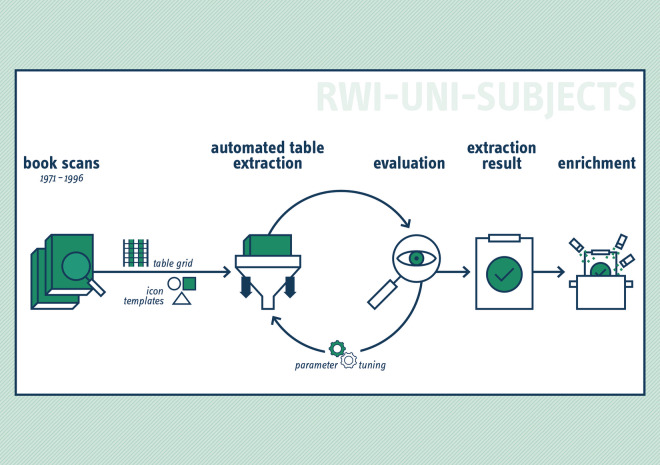
Overview of the extraction process and enrichment of data.

A three-step process was used to compile the dataset (see Fig. 3). First, the study guides titled “Study and Career Choice” were scanned to create digital copies of the books. The second step involved automated table extraction from the scans, including evaluation and repeated adjustments of parameters to optimize performance. Finally, the extracted data were enriched with official administrative classifications and codes to ensure consistency and facilitate further analysis.

### Book scans

**Fig. 4:**
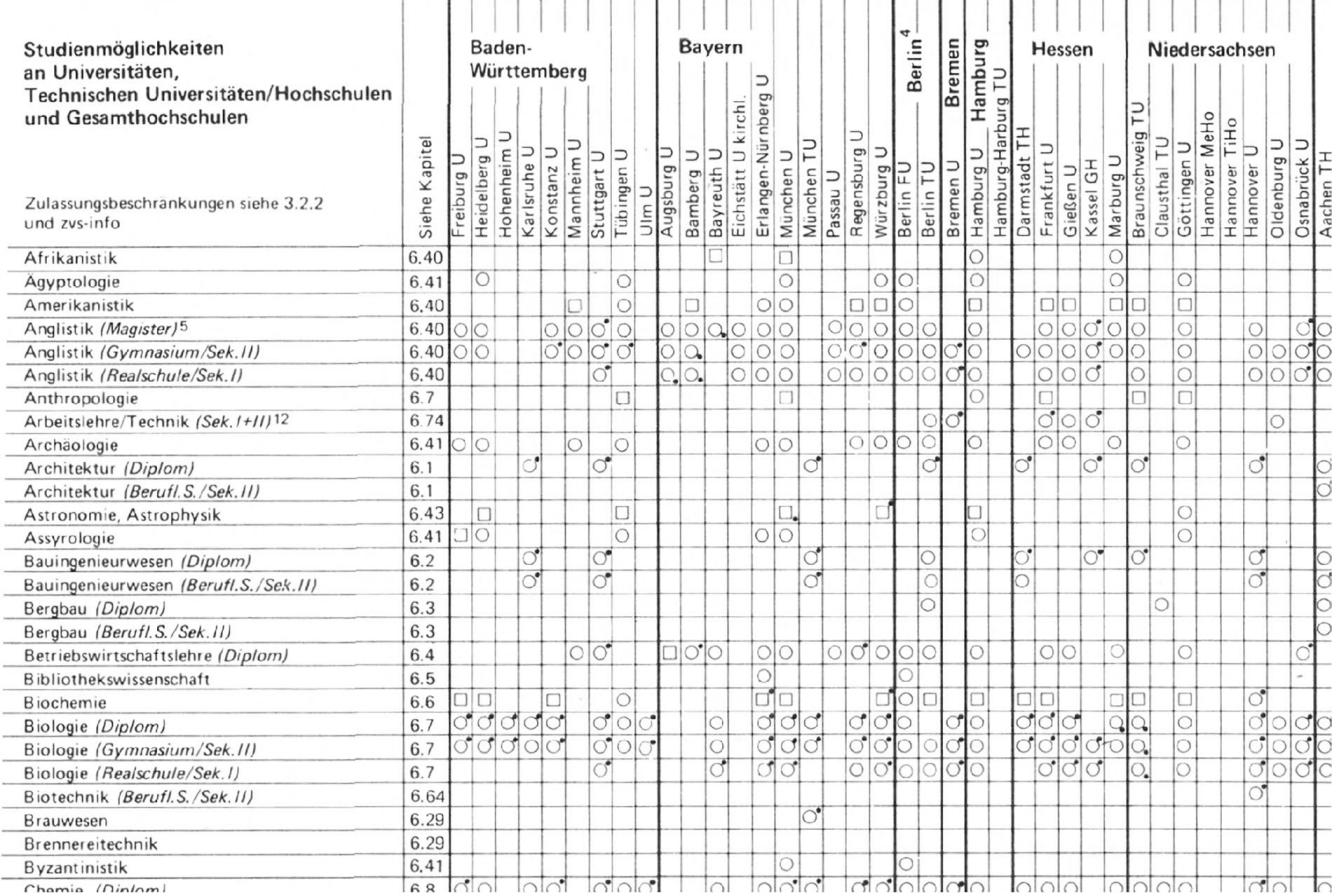
Excerpt of a scan providing information on subjects at universities in 1980^13^. Note: Excerpt of a sample page providing the table of subjects at universities of the year 1980, based on Bock (1980)^13^. The structure of these tables has been standardized throughout the years from 1971 to 1996. Each book includes several pages of tables for universities and other higher education institutions.

All pages of the official study guides were scanned to serve as the primary input for the extraction process. Given the large number of study subjects and institutions covered, each book contained tables that spanned up to 16 pages. An example of an extract of a table showing the availability of study paths in Germany in the year 1980 is shown in Fig. 4. For copyright reasons, no further scans of the tables can be provided. In each table, rows represent academic subjects, while columns correspond to higher education institutions. Each cell is either empty, indicating that the specific subject is not available at the specific higher education institution, or it contains one of various icons (e.g., circles, triangles, or rectangles). These icons represent the availability and mode of the program, such as full-time, part-time, or programs that start exclusively in a specific term.

### Automated table extraction and evaluation

Manually transcribing the tables would have been highly time-consuming and prone to errors. To automate this process, we developed a customizable extraction algorithm^32^ that combines existing computer vision methods. The algorithm is divided into two parts: *i)* extraction of the table grid and *ii)* detection of icons within the table’s cells.

i) To extract the table grid, we used the *OpenCV* library^40^ to identify horizontal and vertical lines within the scanned images. We defined filters for both horizontal and vertical lines, adjusting parameters such as minimum line width, height, and the distance between lines to accommodate variations in table styles across different years. The lines were ordered by their pixel coordinates, and each pair of neighboring lines represented a row or column in the table.
ii) *Template matching*^40^ is a technique for identifying regions in an image that match a predefined template. We applied this method to detect the icons within the cells. The algorithm slides the icon template over the image and calculates a similarity score. If the score exceeds a predefined threshold (between 0.70 and 0.85 – depending on scan quality), the icon is identified and assigned a numerical code. For each cell, only the icon with the highest similarity score was retained, while empty cells were left blank.

Throughout the extraction process, the results were continuously evaluated by manually reviewing interim results. The iterative process required fine-tuning of parameters regarding the grid recognition and template matching (as illustrated in Fig. 3). Axis labels representing subjects and institutions were recorded separately and subsequently reintegrated into the final extraction results. Finally, tables spanning multiple pages were concatenated into a single, cohesive dataset.

### Extraction results and enrichment

We enriched the extraction results by incorporating additional data and harmonizing key variables. The names for higher education institutions were manually adjusted to correct for spelling errors, removal of duplicates, and harmonization of inconsistent naming conventions over time. Also, the administrative code for each HEI was added as provided by the German Federal Statistical Office (Destatis)^41^. Similarly, subject names were harmonized. Each subject was classified according to the German academic coding system, which distinguishes 276 subjects (e.g., business informatics), grouped into 64 subject areas (e.g., computer science) and 9 subject groups (e.g., engineering). The respective administrative codes were also added based on Destatis^42^.

To improve the international usability of the dataset, we translated all variables into English and also matched the subject name to the corresponding international ISCED-F codes. Regarding the translations, all available German terms referring to subjects, subject areas, and subject groups were translated into English using an official mapping list provided by Destatis^43^. However, in some cases, the Destatis_subject field could contain missing values due to ambiguous matches for the subject. To address this issue, we additionally translated all original terms extracted from the study guides (subject) using the pre-trained large language model MarianMT^44^ (Helsinki-NLP/opus-mt-de-en^45^). We fine-tuned the model using the official Destatis translations to align the machine-translated subject titles as closely as possible with the Destatis terminology.

In addition, we included ISCED-F codes in our dataset. ISCED-F is the *“International Standard Classification of Education: Fields of Education and Training 2013”*, an internationally comparable classification on fields of education and training provided by UNESCO^46^. Although no official mapping from German Destatis to ISCED-F codes exists, most of the subjects can be matched unambiguously. However, in some rare cases, particularly in interdisciplinary programs, classifications can vary even across higher education institutions. In such cases, we applied the best possible approximation. For instance, the Technical University Darmstadt assigns two ISCED-F codes for Industrial Engineering *(Wirtschaftsingenieurwesen)*: 041 – Business and Administration (when the majority of courses are in business or economics) or 071 – Engineering and Engineering Trades (when the majority of courses are technical)^47^.

Then, data on each institution’s location (location_name) was added. To enable easy linkage with regional administrative and survey data, we further enriched our dataset by also adding the administrative municipality codes (BKG_municipality_code) of the Federal Agency for Cartography and Geodesy (BKG)^48^ as of 2013 that are commonly used in other datasets.

## Data Record

RWI-UNI-SUBJECTS^1^ is available at 10.7807/studi:buch:suf:v1 as a single file in two formats: .csv and Stata’s .dta, ensuring broad compatibility with various tools and programming languages. The dataset contains detailed information on the availability of study programs at German higher education institutions between the years 1971 and 1996.

Table 1 outlines the core data directly extracted from the study guides, including the names of the institutions and study programs as well as program details. Additional variables created in the data processing phase, for example administrative codes for fields, institutions, and districts, are shown in Table 2. Thereby, the resulting dataset can be efficiently linked with external administrative datasets and survey data. Overall, RWI-UNI-SUBJECTS^1^ covers 105,307 study programs between 1971 and 1996 and thereby provides a robust resource for research in the areas such as education, innovation, economic development, or the history of various subjects in Germany.

**Table 1:** Overview of variables extracted from the study guides.

Variable name	Description
year	Publication year of the study guide and thereby observation (1971 until 1996)
type	Institutional type, e.g., *university* or *university of applied sciences*
hei_name	Institution’s name as provided by the guide
subject	Original study program name as provided by the guide
study_type	Type of study mode (e.g., *full-time study* or *part-time study*)

**Table 2:** Overview of the enrichment variables.

Variable name	Description
Destatis_hei_number	Institution’s code based on Destatis (2022)^41^
Destatis_hei_name	Institution’s name based on Destatis (2022)^41^
Destatis_hei_name_last	Last previous institution’s name based on to Destatis (2022)^41^ in case of observed name changes
exact_hei_name	Equal to 1 if the institution’s name, as provided by the guide, refers to an exact institution, and 0 if it refers to a location-level aggregation
hei_change	Categorical Variable indicating changes in the institution (e.g., merging with another institution)
Destatis_subject	Study program based on Destatis (2023)^42^
Destatis_subject_area	Subject area based on Destatis (2023)^42^
Destatis_subject_group	Subject group based on Destatis (2023)^42^
Destatis_subject_code	Subject code based on Destatis (2023)^42^
Destatis_subject_area_code	Subject area code based on Destatis (2023)^42^
Destatis_subject_group_code	Subject group code based on Destatis (2023)^42^
location_name	Location (city) of the institution
BKG_municipality_code	Municipality code as of December 31, 2013^48^
ISCED_detailed_field	Detailed field classification based on ISCED-F (2013)^46^
ISCED_narrow_field	Narrow field classification based on ISCED-F (2013)^46^
ISCED_broad_field	Broad field classification based on ISCED-F (2013)^46^
ISCED_detailed_field_code	Detailed field code based on ISCED-F (2013)^46^
ISCED_narrow_field_code	Narrow field code based on ISCED-F (2013)^46^
ISCED_broad_field_code	Broad field code based on ISCED-F (2013)^46^
subject_EN	English translation of the original subject
Destatis_subject_EN	English translation of the subject based on Destatis (2023)^43^
Destatis_subject_area_EN	English translation of the subject area based on Destatis (2023)^43^
Destatis_subject_group_EN	English translation of the subject group based on Destatis (2023)^43^
author	The individual or group responsible for writing the study guide
commissioning_body	Exclusively the “Bund-Länder-Kommission für Bildungsplanung und Forschungsförderung (BLK)” in this dataset
title	Full title of the study guide as it appears on the publication
publisher	The publishing institution responsible for the production and distribution of the study guide

### Missing values

While the dataset is largely complete, a small proportion of the enriched variables could not be fully assigned. This leads to a small number of missing values.

i) *Destatis subject matching:* All observations in the dataset have been successfully categorized at the broadest Destatis level, ensuring complete coverage at this classification tier (Destatis_subject_group). At more detailed levels, only a small share of observations could not be matched: 1.49% remain unmatched at the Destatis_subject level, and 0.33% at the Destatis_subject_area level. In these cases, the corresponding codes (Destatis_subject_code and Destatis_subject_area_code) are then also missing. These unmatched cases typically involve subjects that are difficult to categorize under the Destatis (2022)^41^ classification, such as *museum studies* and *crystallography*.
ii) *Institutional matching:* Regarding the names and codes of institutions, 9.90% are missing (Destatis_hei_number and Destatis_hei_name). The vast majority of these unmatched cases (99.96%) result from the fact that until 1985, universities of applied sciences were listed in the study guides only at the location level. Thus, we can identify the study programs offered in a given location during this period, but it is not possible to determine the *exact institution* providing them. In these entries, the variable exact_hei_name is set to 0. The remaining 0.04% of unmatched cases are due to two specific institution names that could not be clearly linked to any official Destatis code and name. For these two institutions, location information and the institution names from the study guides are available, but no official identifiers could be assigned. Additionally, the variable Destatis_hei_name_last contains missing values by design: it is only filled when a change in the institutional name occurred, which applies to 3.33% of all observations.

## Technical Validation

To ensure the integrity and technical validity of the RWI-UNI-SUBJECTS^1^ dataset, we implemented a series of rigorous validation procedures throughout the extraction and processing stages (see Fig. 3).

### Data source integrity

In Germany, career counseling is organized and overseen by the Federal Employment Agency, which holds the legal mandate and state monopoly for providing authoritative information on educational and vocational opportunities^49^. As part of this responsibility, the agency has annually published the study guides since 1971. Until the mid-1990s, these guides served as the primary reference for students and counselors. The guides were compiled under supervision of the Commission for Educational Planning and Research Promotion (BLK), ensuring high standards of accuracy, completeness and consistency^4^. As the sole authorized source of such information during the period covered, these publications provide a solid and reliable foundation for our dataset.

### Validation of extraction algorithm

The scans used for the automated extraction exhibited varying levels of quality. To mitigate the potential impact of scan quality on the extraction process, we visualized interim results, such as detected columns, rows, and items, and compared them to the original data. Additionally, we manually checked randomly selected rows and columns for any errors. Overall, in an iterative process including visual and manual evaluation loops, we refined the extraction algorithm several times in response to identified errors.

### Consistency checks

In addition to the initial extraction validation, we conducted extensive checks for consistency and integrity. The subjects offered by each university were plotted over time, and all resulting visualizations were manually reviewed for plausibility. Any significant fluctuations in the data were flagged for further investigation. When discrepancies were found, the original and generated datasets were manually cross-checked, and the parameters of the extraction algorithm were adjusted accordingly.

### Code matching

To enhance the dataset’s analytical utility and facilitate integration with external data sources, we enriched the original records with official classifications and standardized codes. These include identifiers for higher education institutions and study programs, both provided by the Federal Statistical Office^41,42^, as well as municipality codes provided by the Federal Agency for Cartography and Geodesy^48^. Integrating these codes into RWI-UNI-SUBJECTS^1^ ensures a consistent alignment with national classifications and provides a framework for linking the dataset to existing administrative or survey data.

### Error rate

Automated extractions generally yield lower error rates than manual data entry. This is particularly true for large-scale datasets due to reduced human fatigue and consistency in pattern recognition^50^. To assess the quality of our extraction process, we conducted a systematic validation by manually checking randomly selected samples from all years that comprised 5.8 % of the entire dataset. The observed error rate across these samples varied from 0 % to 1.27 % across years, largely depending on the variations in scan quality. Based on this review, we estimate an extrapolated average error rate of 0.2 %, meaning that roughly one in every five hundred cells may contain an error. Details on the final review are described in Hertweck *et al*.^1^.

## Data Availability

The dataset is publicly available through the Research Data Centre Ruhr (FDZ Ruhr) at 10.7807/studi:buch:suf:v1.

## Code availability

The code is publicly available on https://github.com/Boshl/RWI-UNI-SUBJECTS.
